# Neutrophil-Mediated Lung Injury Both via TLR2-Dependent Production of IL-1α and IL-12 p40, and TLR2-Independent CARDS Toxin after Mycoplasma pneumoniae Infection in Mice

**DOI:** 10.1128/spectrum.01588-21

**Published:** 2021-12-22

**Authors:** Shigeyuki Tamiya, Eisuke Yoshikawa, Monami Ogura, Etsushi Kuroda, Koichiro Suzuki, Yasuo Yoshioka

**Affiliations:** a Laboratory of Nano-Design for Innovative Drug Development, Graduate School of Pharmaceutical Sciences, Osaka University, Suita, Osaka, Japan; b Vaccine Creation Group, BIKEN Innovative Vaccine Research Alliance Laboratories, Research Institute for Microbial Diseases, Osaka University, Suita, Osaka, Japan; c Department of Immunology and Medical Zoology, Hyogo College of Medicine, Nishinomiya, Hyogo, Japan; d The Research Foundation for Microbial Diseases of Osaka University, Suita, Osaka, Japan; e Vaccine Creation Group, BIKEN Innovative Vaccine Research Alliance Laboratories, Institute for Open and Transdisciplinary Research Initiatives, Osaka University, Suita, Osaka, Japan; f Global Center for Medical Engineering and Informatics, Osaka University, Suita, Osaka, Japan; National Institutes of Health

**Keywords:** alveolar macrophage, infection, inflammation, lung injury, *Mycoplasma pneumoniae*, neutrophils, toxin

## Abstract

Mycoplasma pneumoniae (Mp) residing extracellularly in the respiratory tract is the primary cause of bacterial community-acquired pneumonia in humans. However, the detailed pathological mechanism of Mp infection, especially inflammation in the lung, remains unclear. This study examined the role of the neutrophils in the inflammation of Mp-induced pneumonia in mice and the mechanism of neutrophil infiltration into the lungs in the Mp-induced pneumonia. We observed massive infiltration of neutrophils in the bronchoalveolar lavage fluid (BALF) and lung injury after the Mp challenge. The neutrophils were shown to contribute to lung injury in Mp pneumonia but were not involved in eliminating Mp, suggesting that neutrophils are detrimental to the host in Mp pneumonia. Mp also induced the production of inflammatory cytokines and chemokines in the BALF in a toll-like receptor 2 (TLR2)-dependent manner. Particularly, both interleukin (IL)-1α and IL-12 p40 played a crucial role in neutrophil infiltration into the BALF in a coordinated manner. Both IL-1α and IL-12 p40 were released from the alveolar macrophages depending on the TLR2 and reactive oxygen species. In addition, the community-acquired respiratory distress syndrome (CARDS) toxin from Mp were found to induce neutrophil infiltration into BALF in a TLR2-independent and IL-1α-dependent manner. Collectively, the TLR2-dependent production of both IL-1α and IL-12 p40, and CARDS toxin have been elucidated to play an important role in neutrophil infiltration into the lungs subsequently leading to the lung injury upon Mp infection in mice. These data will aid in the development of therapeutics and vaccines for Mp pneumonia.

**IMPORTANCE** Although Mp-induced pneumonia is usually a self-limiting disease, refractory life-threatening pneumonia is often induced. In addition, the development of alternative therapeutic strategies for Mp is expected because of the emergence of antibiotic-resistant Mp. However, the lack of knowledge regarding the pathogenesis of Mp-induced pneumonia, especially inflammation upon the Mp infection, makes it tedious to design novel therapeutics and vaccines. For example, although neutrophil infiltration is widely recognized as one of the characteristics of Mp-induced pneumonia, the precise role of neutrophils in the aggravation of Mp pneumonia remains unclear. This study showed that the infiltration of neutrophils in the lungs is detrimental to the host in Mp-induced pneumonia in mice. Furthermore, the TLR2-dependent IL-1α and IL-12 p40 production, and CARDS toxin play important roles in neutrophil infiltration into the lung, following lung injury. Our findings apply to the rational design of novel therapeutics and vaccines against Mp.

## INTRODUCTION

Mycoplasma pneumoniae (Mp) resides extracellularly in the upper and lower respiratory tracts, often inducing tracheobronchitis in humans (1, 2). The most serious clinical symptom induced by Mp infection is pneumonia, and recent reports have shown Mp to be the primary cause of the bacterial-associated community-acquired pneumonia in the United States, especially in children (3). Although Mp-induced pneumonia is usually a self-limiting disease, refractory life-threatening pneumonia is sometimes induced. Mp infection also exacerbates asthma and immune-mediated complications (1, 4, 5). Although macrolide antibiotics are recommended as the first-line treatment for Mp pneumonia (6), they have been reported to develop macrolide-resistant Mp strains worldwide, especially in Asia (7, 8). Therefore, there is an urgent need to develop alternative therapeutic strategies for preventing the incidence of severe Mp pneumonia.

Although immune responses are caused in the lungs after Mp infection to eliminate Mp, the excessive production of inflammatory cytokines and chemokines skews the host response to hyperinflammation with an exaggerated tissue injury (1, 2, 9). Therefore, aggravating Mp pneumonia culminates from the excessive host immune responses against Mp rather than direct tissue injury by Mp (1, 2). Mycoplasma pulmonis, a natural pathogen for rodents, have been used as a model pathogen in mice and rats to understand the chronic inflammation mechanism of Mp in humans. Several studies have reported the contribution of Mycoplasma pulmonis-specific T cells such as the Th1, Th2, and Th17 cells to the Mycoplasma pulmonis-induced pathogenesis (10, 11), indicating the possibility that these T cells contribute to the Mp-induced chronic inflammation in humans. However, the precise mechanism of acute inflammation after Mp infection remains unclear.

In general, neutrophils function as the first line of innate immune defense against pathogens but are the principal cellular responders to acute inflammation, and excessive activation leads to tissue injury. Neutrophil infiltration is widely recognized as a characteristic of Mp-induced pneumonia (12–14). For example, Guo et al. showed that the percentage of neutrophils and T cells in the bronchoalveolar lavage fluid (BALF) is increased in children with severe Mp pneumonia compared to the mild cases (12), indicating that neutrophils in the BALF may be critical for the inflammatory response in acute and severe Mp pneumonia. Therefore, limiting neutrophil infiltration into the lungs might be a way to manage the severity of Mp-induced pneumonia. However, the precise role of neutrophils in aggravating Mp pneumonia and eliminating Mp remains unclear.

Ciliated epithelial cells and alveolar macrophages in the respiratory tract secrete cytokines and chemokines upon stimulation by Mp, leading to inflammatory responses (1, 2, 15). Some pathogenic factors produced by Mp are thought to induce this immune response (15). For example, Mp possesses membrane-bound lipoproteins such as the diacyl lipoprotein and triacyl lipoprotein, which act as toll-like receptor 2 (TLR2) ligands (15). Several studies have shown that Mp induces cytokines and mucin from the macrophages and epithelial cells via TLR2 *in vitro*, and TLR2 is essential for clearing Mp by inducing mucin production in the lungs of mice (16–18). However, the contribution of TLR2 to inflammatory responses, such as neutrophil infiltration in Mp infection, remains elusive.

Also, the community-acquired respiratory distress syndrome (CARDS) toxin produced from Mp has attracted a lot of attention as a pathogenic factor (19–22). The CARDS toxin has ADP-ribosylating activity and vacuolating activity with the capacity to bind to the airway epithelial cells via the surfactant protein A and annexin A2 (19–21). The amount of CARDS toxin increases during Mp infection in humans and experimental animals (21, 23, 24) and the amount of CARDS toxin in BALF is positively correlated with the severity of the pulmonary disease in mice (22). Furthermore, a clinical study has revealed that the high co-expression of tumor necrosis factor-α (TNF-α) and CARDS toxin in BALF is a good diagnostic biomarker (25). Recombinant CARDS toxin (rCARDS toxin) promotes the release of the inflammatory cytokines such as interleukin (IL)-1β, IL-6, and TNF-α from the airway epithelial cells and macrophages *in vitro* (26) and the rCARDS toxin induces inflammatory responses in the lungs of mice and nonhuman primates (27–29). Hardy et al. have clearly showed that the rCARDS toxin induces the release of inflammatory cytokines and chemokines, the vacuolization and cytotoxicity of epithelial cells, and a robust lymphocyte infiltration in the lungs of mice and nonhuman primates (27), indicating the possibility that the CARDS toxin plays a fundamentally important role in the pathogenesis of Mp infection. However, there is no convincing evidence of the CARDS toxin contributing to inflammatory responses, such as the neutrophil infiltration during Mp infection *in vivo*.

This study examined the role of neutrophils in the acute inflammation of Mp-induced pneumonia and the mechanism of neutrophil infiltration into the BALF in Mp-induced pneumonia.

## RESULTS

### Early phase inflammation caused by the Mp challenge.

**FIG 1:**
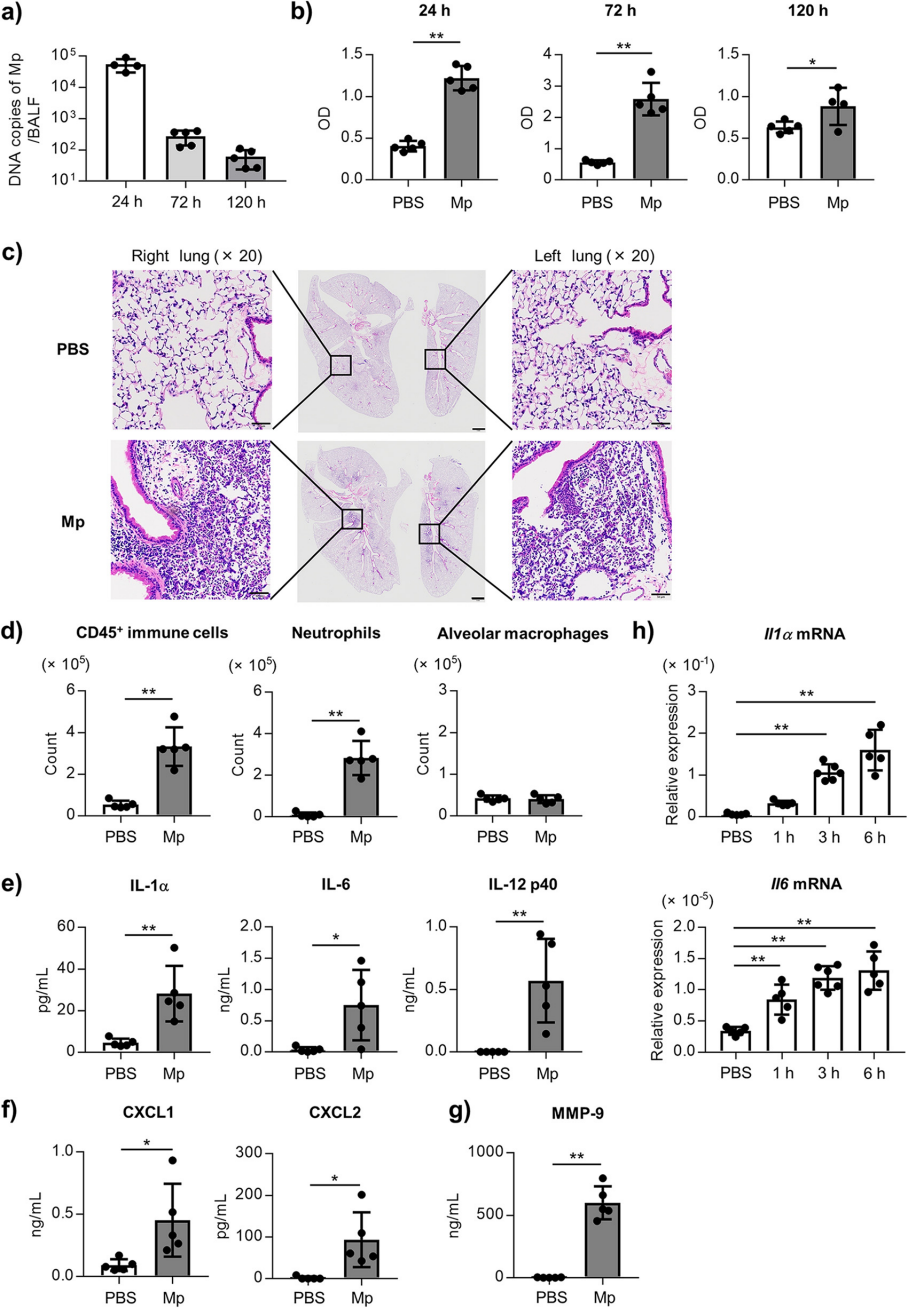
The lung injury and inflammation after the Mp challenge. The mice were challenged intranasally with Mp (6 × 10^7^ CFU). As a control, the mice were treated with PBS intranasally. (a, b) At 24 h, 72 h, and 120 h after the Mp challenge, (a) the DNA copies of Mp in BALF were measured by real-time PCR and (b) the level of LDH in the BALF was measured. (c) The lung pathology was evaluated by H&E staining 24 h after the Mp challenge. Middle panel: scale bars indicated 1 mm; left panel and right panel: scale bars indicated 50 μm. (d–g) At 24 h after the Mp challenge, (d) the numbers of CD45^+^ immune cells, neutrophils (CD45^+^ Ly6G^+^ CD11b^+^ Siglec-F^–^), and alveolar macrophages (CD45^+^ Ly6G^–^ CD11c^+^ Siglec-F^+^) in the BALF were evaluated by flow cytometry, (e) the levels of IL-1α, IL-6, and IL-12 p40 in BALF, (f) the levels of CXCL1 and CXCL2 in BALF, and (g) the level of MMP-9 in the BALF were measured by ELISA. (h) The relative expression levels of the *Il1α* and *Il6* mRNA normalized to *GAPDH* in the BALF at indicated time points after the Mp challenge were measured by real-time PCR. (a–h) Each experiment was performed more than twice. (a, b, d–h) Data are shown as means ± SD. (a, b) *n *= 4–5; (d–g) *n *= 5; (h) *n *= 5–6. *, *P* < 0.05; **, *P* < 0.01 as indicated by (b, d–g) Student's *t* test and (h) Dunnett's test. See also Fig. S1–S4.

To evaluate lung injury in the early phase of Mp infection, the number of Mp and the level of lactate dehydrogenase (LDH) as a marker of tissue injury, were evaluated in the BALF after intranasal challenge with Mp. The number of Mp in the BALF was highest at 24 h after the Mp challenge and gradually decreased but remained detectable up to 120 h (Fig. 1a). The level of LDH in the BALF was significantly higher at 24, 72, and 120 h after the Mp challenge compared to the PBS-treated control mice (Fig. 1b). We confirmed that this LDH level is comparable to the LDH level after the intranasal treatment of lipopolysaccharide (Fig. S1), which is generally used to induce lung injury (30, 31). In addition, we observed massive neutrophil infiltration in the lungs 24 h after the Mp challenge using hematoxylin and eosin (H&E) staining (Fig. 1c).

Next, the numbers of CD45^+^ immune cells, neutrophils, and alveolar macrophages in the BALF were measured using flow cytometry (Fig. S2), and the levels of inflammatory cytokines and chemokines in the BALF were assessed by ELISA 24 h after Mp challenge. The numbers of CD45^+^ immune cells and neutrophils in the BALF were significantly increased by the Mp challenge, while there was no change in the number of alveolar macrophages (Fig. 1d). We also observed a significantly higher number of neutrophils in the BALF at 72 h (Fig. S3a), but not 120 h (Fig. S3b), after the Mp challenge. In addition, the levels of inflammatory cytokines, such as IL-1α, IL-6, and IL-12 p40, and chemokines for neutrophil infiltration, such as C-X-C motif chemokine ligand 1 (CXCL1) and CXCL2, in the BALF were significantly increased by the Mp challenge (Fig. 1e and f). The level of matrix metallopeptidase 9 (MMP-9) was also increased by the Mp challenge (Fig. 1g). We did not observe an elevated level of IL-1β in the BALF following Mp challenge (data not shown), while the level of IL-1β in the lung homogenate were significantly increased by the Mp challenge (Fig. S4). The mRNA expression of *IL-1α* and *IL-6* in the BALF also increased at 1 h after the Mp challenge, and further increased with time (Fig. 1h).

### Neutrophils contribute to the lung injury induced by the Mp challenge, but not Mp clearance.

**FIG 2:**
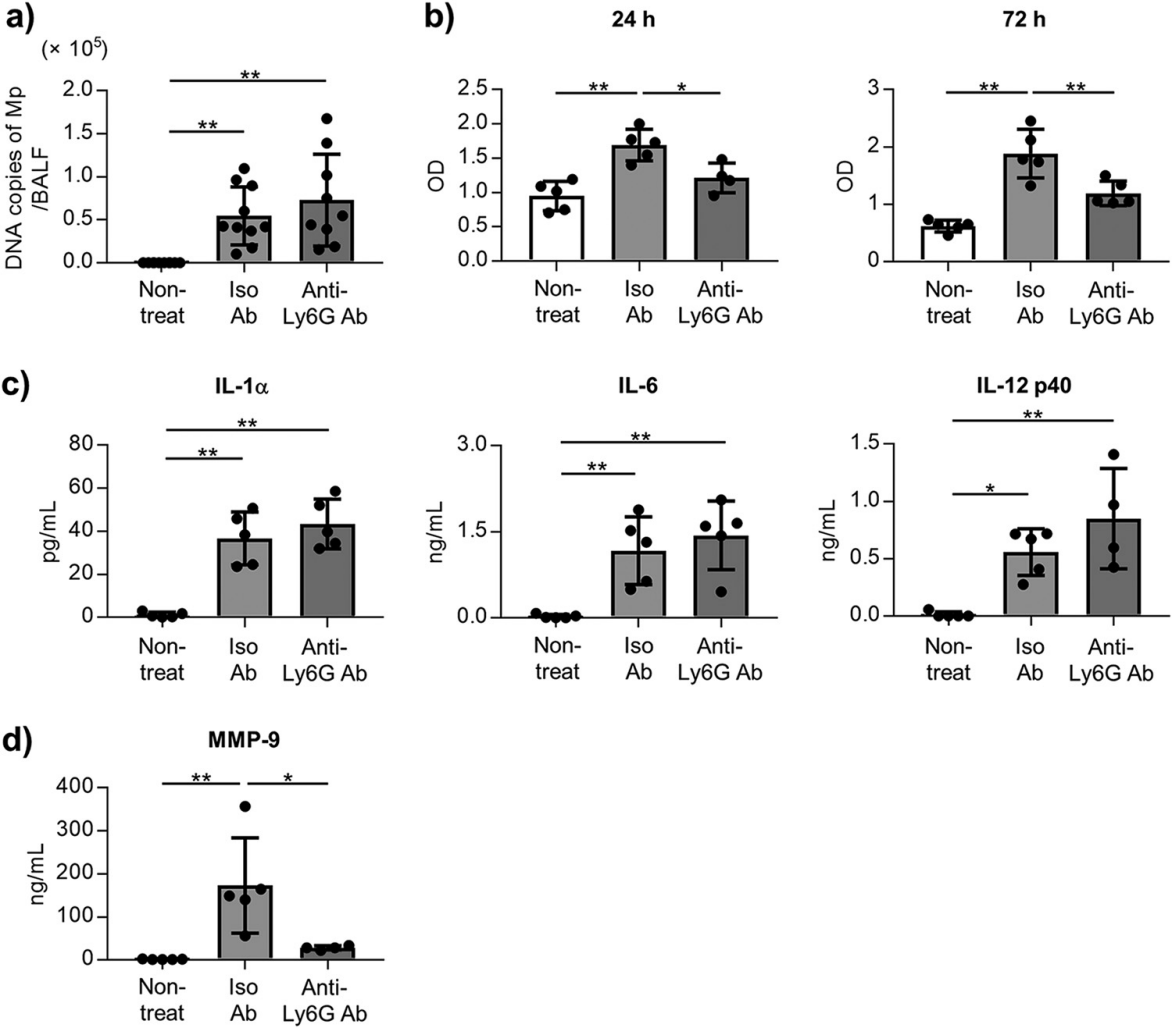
The role of neutrophils in the Mp-mediated lung injury and inflammation. The mice were treated intraperitoneally with an anti-Ly6G antibody (Ab) (100 μg/mouse) or isotype control Ab and challenged with Mp (6 × 10^7^ CFU) on 24 h after Ab treatment. Nontreat group indicated mice treated with PBS intranasally and without Ab treatment. (a) The DNA copies of Mp in the BALF were measured by real-time PCR 24 h after the Mp challenge. (b) At 24 h and 72 h after the Mp challenge, the level of LDH in BALF was measured. (c, d) At 24 h after the Mp challenge, (c) the levels of IL-1α, IL-6, and IL-12 p40 and, (d) the level of MMP-9 in BALF were measured by ELISA. (a–d) Each experiment was performed more than twice. Data are shown as means ± SD. (a) *n* = 9–10; (b–d) *n* = 4–5. (a–d) *, *P* < 0.05; **, *P* < 0.01 as indicated by Tukey’s test. See also Fig. S5.

To determine the contribution of neutrophils to lung injury, we depleted the neutrophils in the mice before the Mp challenge by using an anti-Ly6G antibody (Ab). We confirmed that the number of neutrophils in BALF was significantly decreased after the Mp challenge upon treatment with an anti-Ly6G Ab (Fig. S5). There was no difference in the number of Mp in the BALF 24 h after the Mp challenge between anti-Ly6G Ab-treated mice and isotype control Ab-treated mice (Fig. 2a). In contrast, the levels of LDH in the BALF 24 h and 72 h after Mp challenge in the anti-Ly6G Ab-treated mice were significantly lower than those in the isotype control Ab-treated mice (Fig. 2b). In addition, we did not observe any differences in the levels of IL-1α, IL-6, and IL-12 p40 in the BALF after the Mp challenge between the anti-Ly6G Ab-treated mice and isotype control Ab-treated mice (Fig. 2c). The level of MMP-9 in the BALF from anti-Ly6G Ab-treated mice was lower than that from the isotype control Ab-treated mice (Fig. 2d). These results suggest that neutrophils play a crucial role as the effector cells for lung injury induced by the Mp challenge, but are not involved in the elimination of Mp in the lungs.

### TLR2 contributes to neutrophil infiltration, and cytokine and chemokine production.

**FIG 3:**
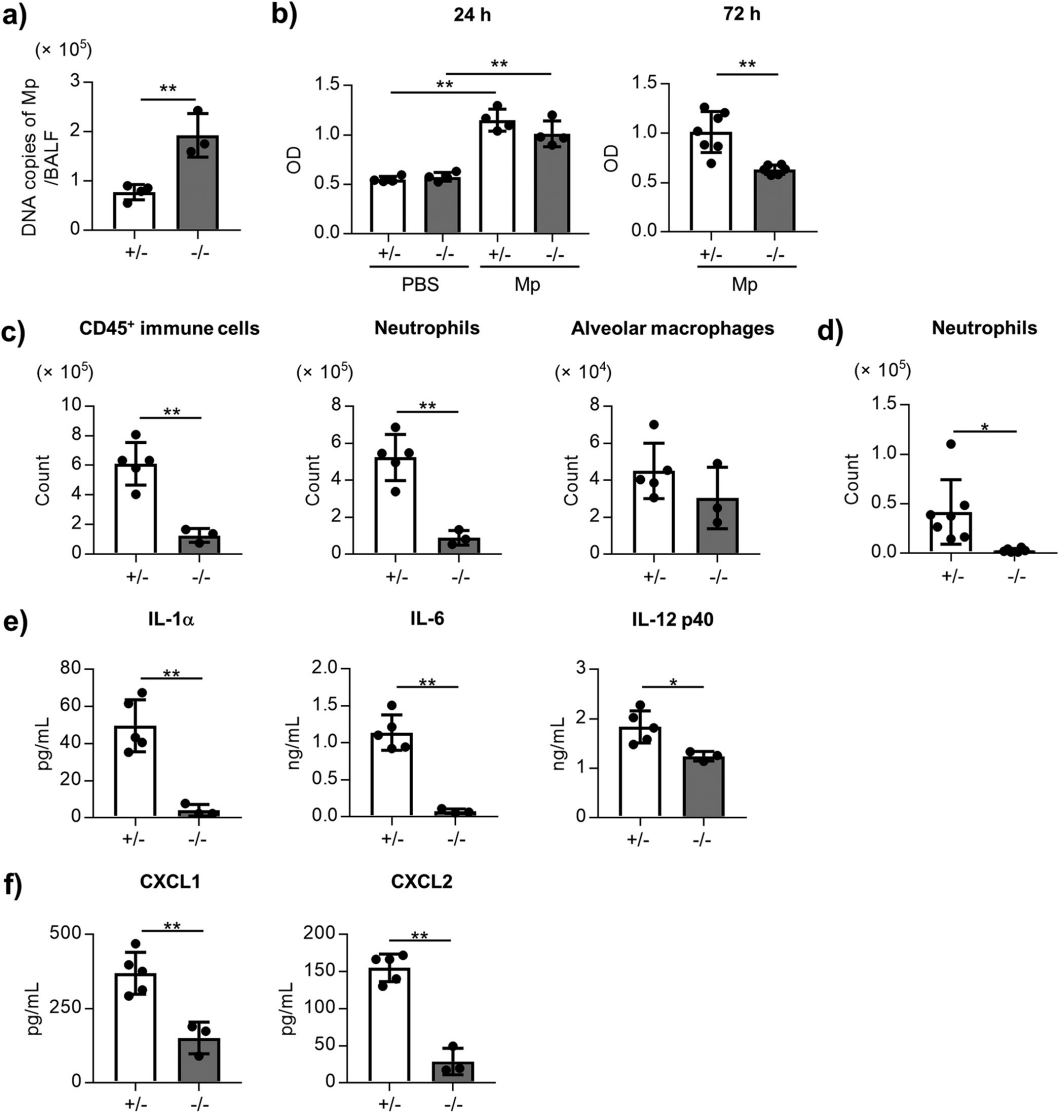
The role of TLR2 in the Mp-mediated lung injury and inflammation. The *TLR2*^+/−^ and *TLR2*^−/−^ mice were challenged intranasally with Mp (6 × 10^7^ CFU). (a) At 24 h after the Mp challenge, the DNA copies of Mp were measured by real-time PCR. (b) At 24 h and 72 h after the Mp challenge, the level of LDH in BALF was measured. (c) At 24 h after the Mp challenge, the numbers of CD45^+^ immune cells, neutrophils (CD45^+^ Ly6G^+^ CD11b^+^ Siglec-F^–^), and alveolar macrophages (CD45^+^ Ly6G^–^ CD11c^+^ Siglec-F^+^) in the BALF were measured by flow cytometry. (d) At 72 h after the Mp challenge, the number of neutrophils in BALF were measured by flow cytometry. (e, f) At 24 h after the Mp challenge, (e) the levels of IL-1α, IL-6, and IL-12 p40 and, (f) the levels of CXCL1 and CXCL2 in the BALF were measured by ELISA. (a–f) Each experiment was performed more than twice. Data are shown as means ± SD. (a) *n *= 3–4; (b) *n *= 4–7; (c) *n *= 3–5; (d) *n *= 6–7; (e–f) *n *= 3–5. *, *P* < 0.05; **, *P* < 0.01 as indicated by (a, b: right panel, c–f) Student's *t* test and (b: left panel) Tukey’s test.

To elucidate the contribution of TLR2 in neutrophil infiltration, we compared the neutrophil infiltration and cytokine production in the BALF after Mp challenge between the homozygous *TLR2*^−/−^ mice and heterozygous *TLR2*^+/−^ mice as controls. The number of Mp in BALF 24 h after Mp challenge in the *TLR2*^−/−^ mice were significantly higher than that in the *TLR2*^+/−^ mice (Fig. 3a). The level of LDH in BALF 72 h after Mp challenge was significantly lower in the *TLR2*^−/−^ mice than *in TLR2*^+/−^ mice, although there was no difference in the level of LDH 24 h after Mp challenge between *TLR2*^+/−^ and *TLR2*^−/−^ mice (Fig. 3b). The numbers of CD45^+^ immune cells and neutrophils in BALF 24 h (Fig. 3c) and 72 h (Fig. 3d) after the Mp challenge were significantly lower in the *TLR2*^−/−^ mice than in the *TLR2*^+/−^ mice. In addition, the levels of IL-1α, IL-6, IL-12 p40, CXCL1, and CXCL2 in the BALF from the *TLR2*^−/−^ mice were significantly lower than those from the *TLR2*^+/−^ mice 24 h after the Mp challenge (Fig. 3e and f). Collectively, TLR2 plays a crucial role in eliminating Mp, in lung injury associated with neutrophil infiltration, and cytokine and chemokine production.

### Both IL-1α and IL-12 p40 contribute to neutrophil infiltration by the Mp challenge.

**FIG 4:**
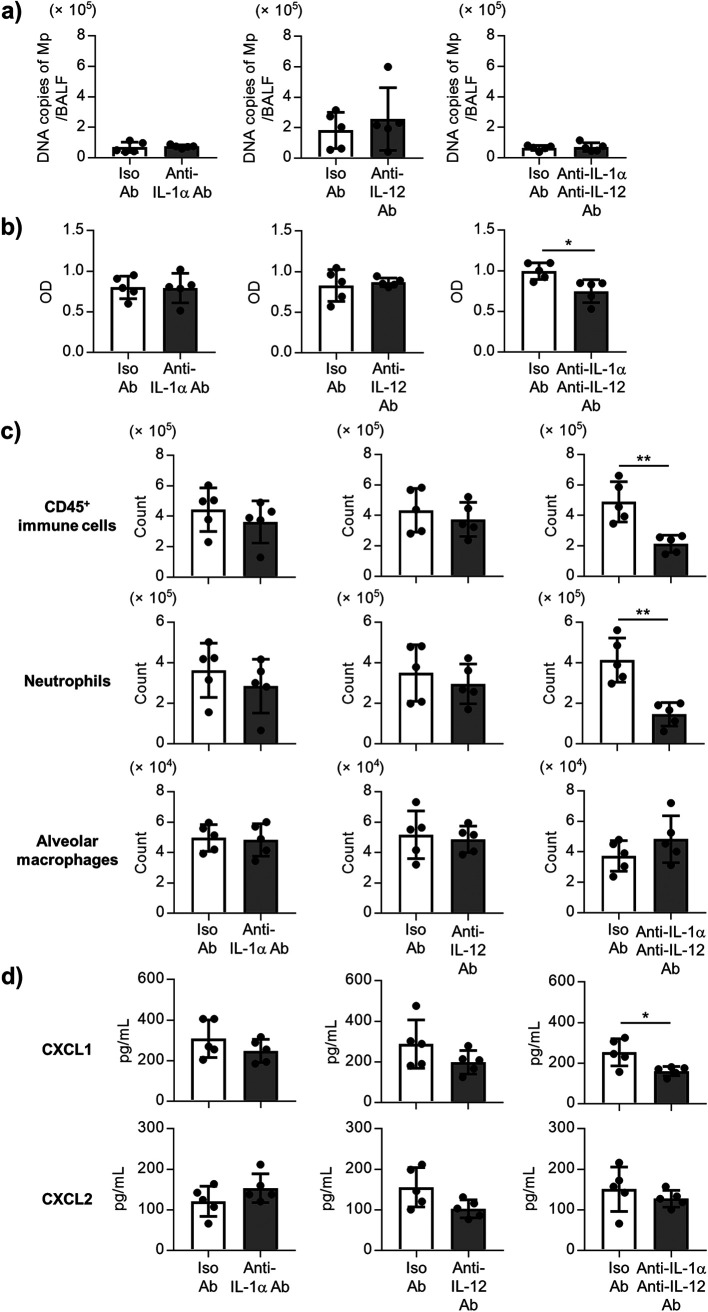
The contribution of IL-1α and IL-12 p40 for the Mp-mediated lung injury and inflammation. The mice were treated intranasally with an anti-IL-1α antibody (Ab) (20 μg/mouse) and/or an anti-IL-12 p40 Ab (50 μg/mouse), or isotype control Ab, and challenged with Mp (6 × 10^7^ CFU) 1 h after Ab treatment. (a) At 24 h after the Mp challenge, the DNA copies of Mp in the BALF were measured by real-time PCR. (b) At 24 h after the Mp challenge, the level of LDH in the BALF was measured. (c) At 24 h after the Mp challenge, the numbers of CD45^+^ immune cells, neutrophils (CD45^+^ Ly6G^+^ CD11b^+^ Siglec-F^–^), and alveolar macrophages (CD45^+^ Ly6G^–^ CD11c^+^ Siglec-F^+^) in the BALF were measured by flow cytometry. (d) At 24 h after the Mp challenge, the levels of CXCL1 and CXCL2 in BALF were measured by ELISA. (a–d) Each experiment was performed more than twice. Data are shown as means ± SD. *n *= 5. *, *P* < 0.05; **, *P* < 0.01 as indicated by Student's *t* test. See also Fig. S6.

Subsequently, the specific cytokines inducing neutrophil infiltration by the Mp challenges were identified with a specific focus on the IL-1α and IL-12 p40. The mice were treated with the neutralizing anti-IL-1α and/or neutralizing anti-IL-12 p40 Abs during the Mp challenge. However, no differences were observed in the number of Mp in the BALF after treatment with the anti-IL-1α Ab, anti-IL-12 p40 Ab, or both anti-IL-1α Ab and anti-IL-12 p40 Ab 24 h after Mp challenge (Fig. 4a). In contrast, the level of LDH in the BALF after the Mp challenge was significantly lower in the mice treated with both anti-IL-1α and anti-IL-12 p40 Abs than in the mice treated with the isotype control Abs, although there was no change in anti-IL-1α Ab-treated mice and anti-IL-12 p40 Ab-treated mice compared to the isotype control Ab-treated mice (Fig. 4b). In addition, the numbers of CD45^+^ immune cells and neutrophils in the BALF from the mice treated with both anti-IL-1α and anti-IL-12 p40 Abs were significantly lower than that in the isotype control Ab-treated mice, although the Ab-treated mice did not show any change (Fig. 4c). At 72 h after the Mp challenge, the same tendency was observed, and the number of neutrophils in the BALF from the mice treated with both anti-IL-1α and anti-IL-12 p40 Abs were significantly lower than those in the isotype control Ab-treated mice (Fig. S6). In addition, the level of CXCL1 in the BALF from the mice treated with both anti-IL-1α and anti-IL-12 p40 Abs was significantly lower than those in the isotype control Ab-treated mice, although the Ab-treated mice did not show any change, while no change was observed in the level of CXCL2 between both anti-IL-1α and anti-IL-12 p40 Ab-treated mice, and isotype control Ab-treated mice (Fig. 4d). These results suggest that both IL-1α and IL-12 p40 contribute to neutrophil infiltration into the lung by the Mp challenge in a coordinated manner.

### Reactive oxygen species (ROS) induces cytokine productions from the alveolar macrophages after Mp treatment *in vitro*.

**FIG 5:**
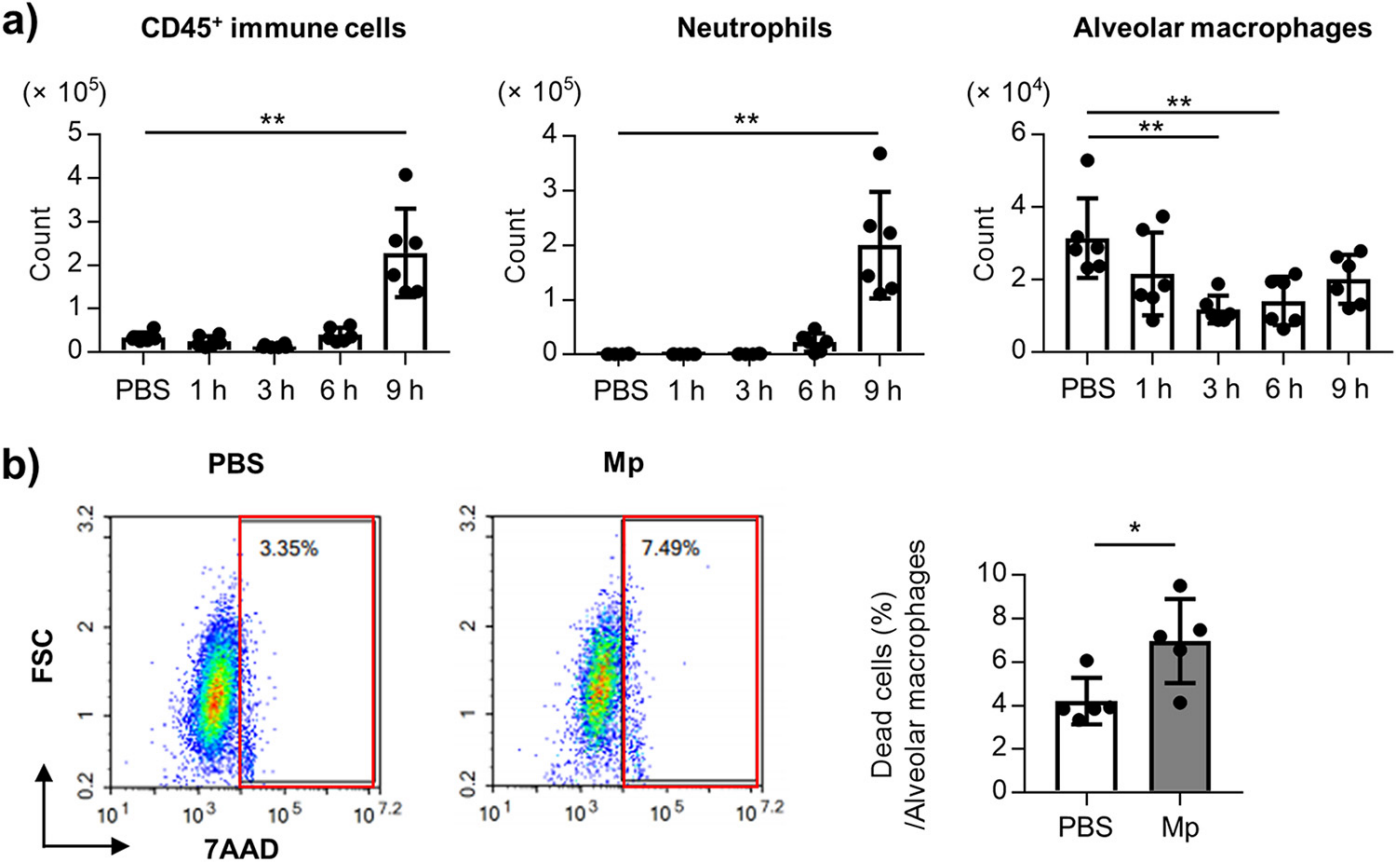
Cell death of the alveolar macrophages after Mp challenge. The mice were challenged intranasally with Mp (6 × 10^7^ CFU). As a control, the mice were treated with PBS intranasally. (a) At 1 h, 3 h, 6 h, and 9 h after the Mp challenge, the numbers of CD45^+^ immune cells, neutrophils (CD45^+^ Ly6G^+^ CD11b^+^ Siglec-F^–^), and alveolar macrophages (CD45^+^ Ly6G^–^ CD11c^+^ Siglec-F^+^) in the BALF were measured by flow cytometry. (b) At 3 h after the Mp challenge, the viability of the alveolar macrophages in the BALF was analyzed by using flow cytometry. The death of the alveolar macrophages was defined as 7AAD^+^ CD45^+^ Ly6G- CD11c^+^ Siglec-F^+^. The left panel showed representative flow cytometry plots of 7AAD^+^ alveolar macrophages. The right panel showed the percentage of 7AAD^+^ alveolar macrophages. (a, b) Each experiment was performed more than twice. Data are shown as means ± SD. (a) *n *= 6; (b) *n *= 5. *, *P* < 0.05; **, *P* < 0.01 as indicated by the (a) Dunnett's test and (b) Student's *t* test.

To investigate the mechanism of cytokine production after the Mp challenge, the numbers of CD45^+^ immune cells, neutrophils, and alveolar macrophages were evaluated in the BALF within 24 h after the Mp challenge. A significantly higher numbers of CD45^+^ immune cells and neutrophils were observed at 9 h, but not at 1, 3, and 6 h after the Mp challenge (Fig. 5a). In contrast, the number of alveolar macrophages was significantly lowered 3 h and 6 h after the Mp challenge than in the control mice (Fig. 5a). In addition, the proportion of the 7-AAD^+^ alveolar macrophages was significantly higher in the Mp-challenged mice than in the control mice (Fig. 5b), indicating that Mp induces the death of the alveolar macrophages.

**FIG 6:**
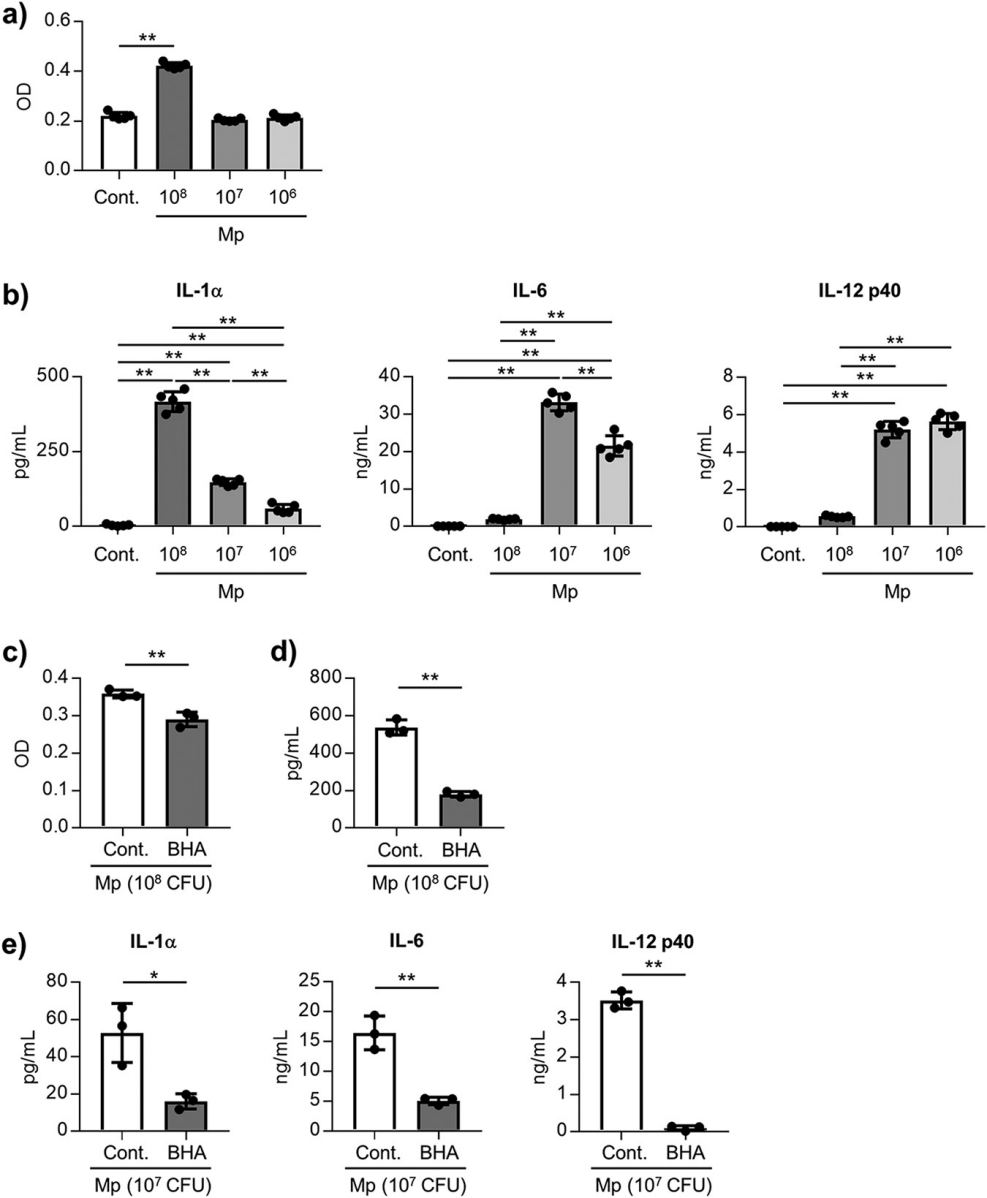
ROS-mediated IL-1α and IL-12 p40 production from the alveolar macrophages. (a, b) The alveolar macrophages were treated with several doses of Mp (1 × 10^8^, 1 × 10^7^, or 1 × 10^6^ CFU/well) for 24 h. (a) The LDH level in the culture supernatant was measured to evaluate the cytotoxic effect of Mp. (b) The levels of IL-1α, IL-6, and IL-12 p40 in the cultured supernatant were measured by ELISA. (c–e) Alveolar macrophages were cultured with Mp (c, d: 1 × 10^8^ CFU/well, e: 1 × 10^7^ CFU/well) for 24 h in the absence or presence of BHA, ROS inhibitor. (c) The LDH level in the culture supernatant was measured to evaluate the cytotoxic effect of Mp. (d) The level of IL-1α in the cultured supernatant was measured by ELISA. (e) The levels of IL-1α, IL-6, and IL-12 p40 in the culture supernatant were measured by ELISA. (a–e) Each experiment was performed more than twice. Data are shown as means ± SD. (a, b) *n *= 5; (c–e) *n *= 3. *, *P* < 0.05; **, *P* < 0.01 as indicated by (a) Dunnett's test, (b) Tukey’s test, and (c–e) Student's *t* test. See also Fig. S7 and S8.

Further investigations were focused on the alveolar macrophages to elucidate the mechanism of cytokine production induced by the Mp challenge. The levels of LDH and cytokines were evaluated in the supernatants 24 h after treatment of the alveolar macrophages from the naive mice with several doses of Mp *in vitro*. Significantly higher levels of LDH were observed in the high-dose Mp-treated cells than in the nontreated cells, although treatment with the middle and low doses of Mp did not induce an increase in the LDH levels (Fig. 6a). We confirmed that about 50% alveolar macrophages died upon high-dose Mp treatment (Fig. S7). Consistent with the results from LDH, the level of IL-1α in the high-dose Mp-treated cells was highest among the Mp-treated groups, while treatment with the middle and low doses of Mp induced significantly higher levels of IL-1α than the nontreated cells (Fig. 6b). In contrast, significantly higher levels of IL-6 and IL-12 p40 were observed in the middle- and low-dose Mp-treated cells than in the high-dose Mp-treated cells and nontreated cells (Fig. 6b). These results suggest that high doses of Mp induce the death of the alveolar macrophages and IL-1α production, while middle and low doses of Mp induce the production of the IL-1α, IL-6, and IL-12 p40.

To elucidate the precise mechanism of cell death and cytokine production induced by Mp in the alveolar macrophages, the contribution of the ROS was examined by using butylated hydroxyanisole (BHA), a ROS inhibitor. The BHA treatment significantly decreased the levels of LDH (Fig. 6c) and IL-1α production (Fig. 6d) induced by high-dose Mp. In addition, treating with a middle dose of Mp was found to significantly decrease the induction of the levels of IL-1α, IL-6, and IL-12 p40 after treatment with BHA (Fig. 6e). The levels of IL-1α, IL-6, and IL-12 p40 confirmed from the alveolar macrophages after stimulation with Pam2CKS4, a TLR2 ligand, were decreased upon treatment with BHA (Fig. S8). These data indicate that ROS caused by Mp induces the death of the alveolar macrophages following IL-1α production in high-dose Mp-treated cells, and induces the IL-1α, IL-6, and IL-12 p40 in the middle-dose Mp-treated cells.

### CARDS toxin involves neutrophil infiltration.

**FIG 7:**
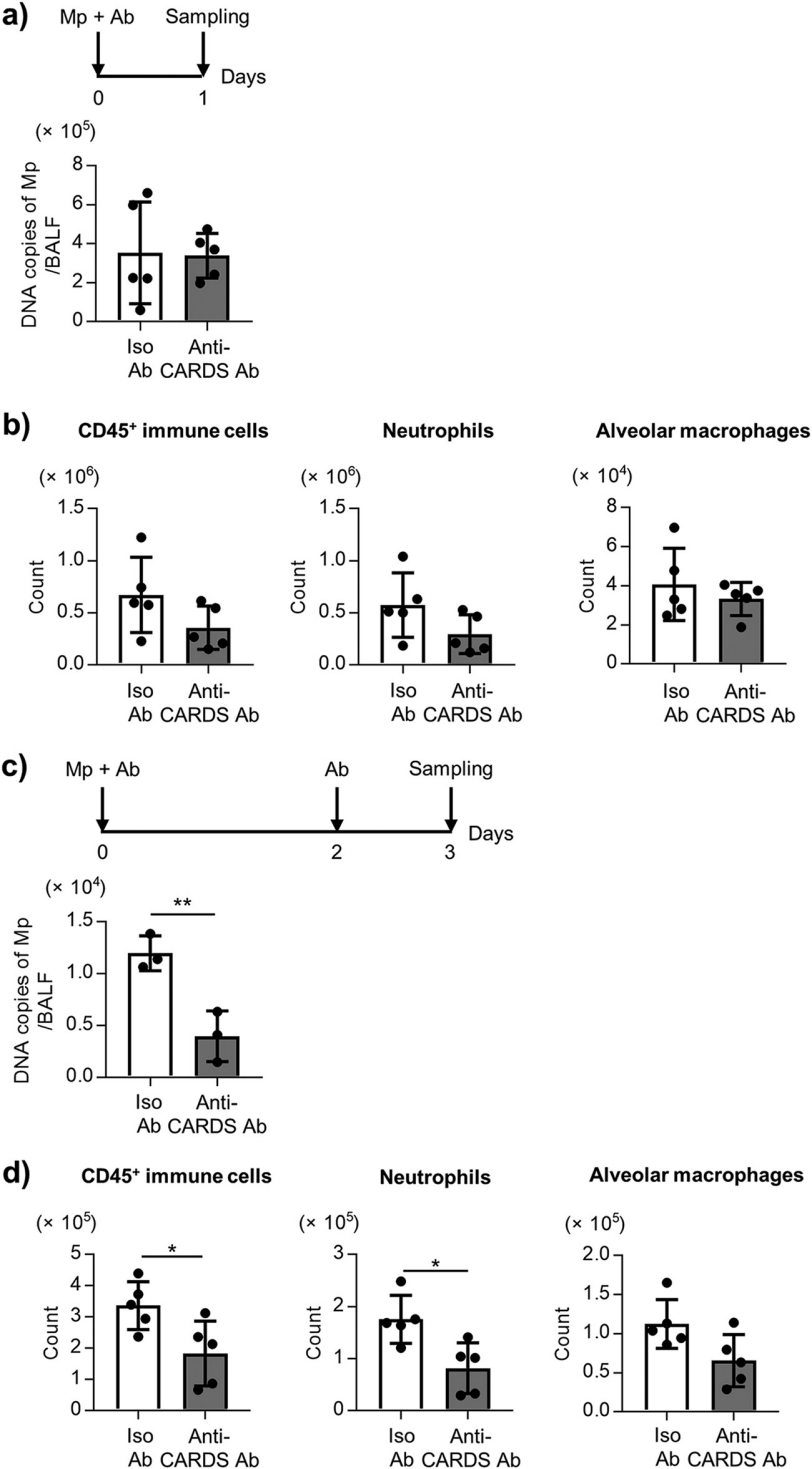
CARDS toxin-dependent Mp persistence and neutrophil infiltration after Mp challenge in mice. (a, b) The mice were challenged intranasally with Mp (6 × 10^7^ CFU) plus anti-CARDS toxin polyclonal antibody (Ab) or isotype control Ab. After 24 h, (a) the DNA copies of Mp in the BALF were measured by real-time PCR, and (b) the numbers of CD45^+^ immune cells, neutrophils (CD45^+^ Ly6G^+^ CD11b^+^ Siglec-F^–^), and alveolar macrophages (CD45^+^ Ly6G^–^ CD11c^+^ Siglec-F^+^) in the BALF were measured by flow cytometry. (c, d) The mice were challenged intranasally with Mp (6 × 10^7^ CFU) plus anti-CARDS toxin Ab or isotype control Ab, and then injected intranasally with anti-CARDS toxin Ab or isotype control Ab again at 48 h after Mp challenge. At 72 h after the Mp challenge, (c) the DNA copies of Mp in the BALF were measured by real-time PCR, and (d) the numbers of CD45^+^ immune cells, neutrophils, and alveolar macrophages in BALF were measured by flow cytometry. (a–d) Each experiment was performed more than twice. Data are shown as means ± SD. (a, b, d) *n *= 5; (c) *n *= 3. (c, d) *, *P* < 0.05; **, *P* < 0.01 as indicated by the Student's *t* test. See also Fig. S9.

To examine the involvement of CARDS toxin in determination of the number of Mp in BALF and neutrophil infiltration into the BALF caused by the Mp challenge, the mice were intranasally challenged with Mp in the presence of anti-CARDS toxin Ab. There was no change in the number of Mp in the BALF (Fig. 7a), and the numbers of CD45^+^ immune cells, neutrophils, and alveolar macrophages in the BALF (Fig. 7b) 24 h after the Mp challenge between the anti-CARDS toxin Ab-treated group and the isotype control Ab-treated group. In contrast, 72 h after the Mp challenge, the number of Mp in the BALF (Fig. 7c) and the numbers of CD45^+^ immune cells and neutrophils in BALF (Fig. 7d) from mice treated with anti-CARDS toxin Ab were significantly lower than those in mice treated with isotype control Ab. In addition, there was no change in the numbers of CD45^+^ immune cells and neutrophils in the BALF from *TLR2*^−/−^ mice 24 h after the Mp challenge between the anti-CARDS toxin Ab-treated group and the isotype control Ab-treated group (Fig. S9). We were unable to examine the effect of anti-CARDS toxin Ab on neutrophil infiltration 72 h after the Mp challenge in *TLR2*^−/−^ mice, because the numbers of neutrophils in BALF were very low (Fig. 3d). These data suggest that the CARDS toxin contributes to the persistence of Mp and neutrophil infiltration after the Mp challenge.

**FIG 8:**
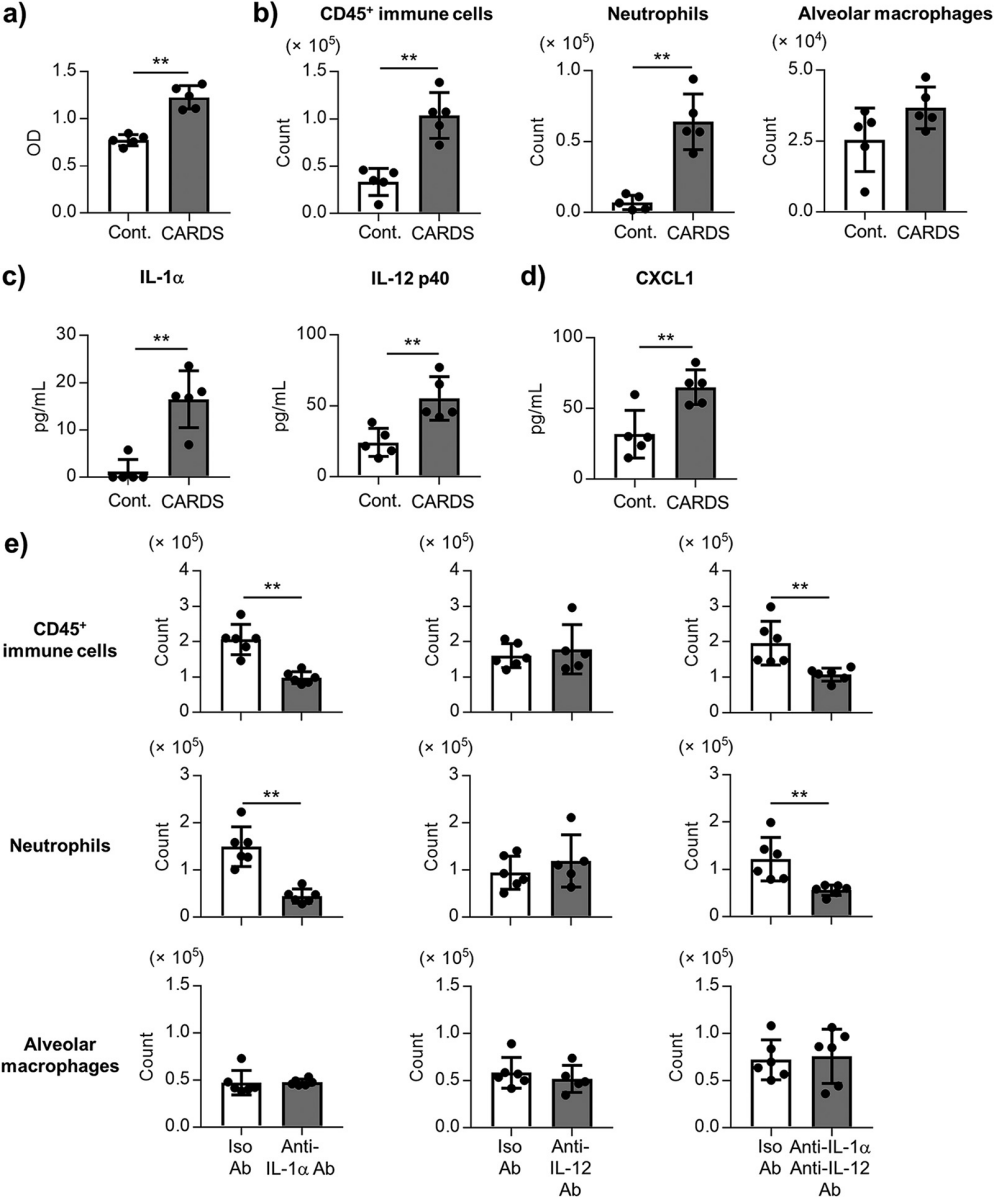
IL-1α-mediated neutrophil infiltration by the CARDS toxin. (a–d) The mice were treated intranasally with the CARDS toxin (50 μg/mouse). At 24 h after the CARDS toxin treatment, (a) the level of LDH in the BALF, (b) the numbers of CD45^+^ immune cells, neutrophils (CD45^+^ Ly6G^+^ CD11b^+^ Siglec-F^–^), and alveolar macrophages (CD45^+^ Ly6G^–^ CD11c^+^ Siglec-F^+^) in the BALF, (c) the levels of IL-1α and IL-12 p40 in the BALF, and (d) the level of CXCL1 in the BALF were measured by (b) flow cytometry and (c, d) ELISA. (e) The mice were treated intranasally with an anti-IL-1α antibody (Ab) (20 μg/mouse) and/or an anti-IL-12 p40 Ab (50 μg/mouse), or isotype control Ab, and then treated intranasally with CARDS toxin (50 μg/mouse) 1 h after Ab treatment. At 24 h after the CARDS toxin treatment, the numbers of CD45^+^ immune cells, neutrophils, and alveolar macrophages in the BALF were measured by flow cytometry. (a–e) Each experiment was performed more than twice. Data are shown as means ± SD. (a–d) *n *= 5; (e) *n *= 5–6. (a–e) **, *P* < 0.01 as indicated by Student's *t* test. See also Fig. S10 and S11.

Furthermore, the mechanism of the CARDS toxin-induced neutrophil infiltration into the lung was investigated using the rCARDS toxin prepared using E. coli. The rCARDS toxin exhibited a vacuolated appearance in the A549 cells *in vitro* (Fig. S10a). The rCARDS toxin-induced IL-1β production in the THP-1 cells, also was inhibited by treating with a specific caspase-1 inhibitor (Fig. S10b). These results suggest that our generated rCARDS toxin has biological activity. When the mice were treated intranasally with the rCARDS toxin, the LDH level in the BALF was significantly elevated by the rCARDS toxin treatment after 24 h (Fig. 8a). The numbers of CD45^+^ immune cells and neutrophils, as well as the levels of IL-1α, IL-12 p40, and CXCL1, were also significantly elevated by the rCARDS toxin treatment (Fig. 8b–d). There was no difference in the numbers of CD45^+^ immune cells and neutrophils 24 h after the rCARDS toxin treatment between the *TLR2*^+/−^ and *TLR2*^−/−^ mice (Fig. S11). Subsequently, the mice were treated intranasally with the anti-IL-1α Ab and/or anti-IL-12 p40 Ab 1 h before treatment with the rCARDS toxin. There was a significant decrease in the numbers of CD45^+^ immune cells and neutrophils in the anti-IL-1α Ab-treated mice and both anti-IL-1α and anti-IL-12 p40 Ab-treated mice compared to those in the isotype control Ab-treated mice 24 h after rCARDS toxin treatment (Fig. 8e). However, there was no difference in the numbers of CD45^+^ immune cells and neutrophils between the anti-IL-12 p40 Ab-treated mice and the isotype control Ab-treated mice (Fig. 8e). These results substantiated that IL-1α contributes to the neutrophil infiltration by the rCARDS toxin.

## DISCUSSION

A significant elevation was observed not only in the number of neutrophils but also in the level of LDH in the BALF after the Mp challenge (Fig. 1, Fig. S1 and S3). Furthermore, the neutrophils were demonstrated to contribute to lung injury and were not found to play a crucial role in eliminating Mp (Fig. 2). A previous report also showed that neutrophils are not important for eliminating Mp in mice (32). These results suggested that the neutrophil infiltrations into the lungs after Mp infection are detrimental to the host. The activation of neutrophils generally releases excessive myeloperoxidase (MPO), MMP-9, and neutrophil elastase (NE), following tissue injury (33). Chen et al. showed that the increased levels of MPO, MMP-9, and NE in the patients with Mp pneumonia are decreased in the convalescent phase (13). Therefore, neutrophil inhibition might be an appropriate approach for managing the Mp pneumonia severity.

Previous reports have shown that the TLR2-dependent mucin production from epithelial cells is crucial for the clearance of Mp and Mycoplasma pulmonis (16, 18, 34). Consistent with this report, TLR2 was found to be important for the clearance of Mp in the BALF (Fig. 3). In contrast, TLR2 was also found to contribute to the infiltration of neutrophils into the BALF and in neutrophil-dependent lung injury (Fig. 3). These results suggested TLR2 acts as a double-edged sword, not only eliminating Mp but also causing tissue injury by inducing neutrophil infiltration. We also showed that the inflammatory cytokines such as IL-1α, IL-6, and IL-12 p40 in Mp-induced pneumonia are dependent on the TLR2 in mice (Fig. 3). In contrast with our results, a previous report showed the *TLR2^−/−^* mice to possess higher levels of cytokines such as IL-6 and TNF-α in BALF after Mycoplasma pulmonis challenge than the wild-type mice (34). Although these conflicting results are not completely understood, the differences in the mycoplasma, Mp and Mycoplasma pulmonis, are suspected to have yielded these contradictory results, which need further investigation in the future.

IL-8 is an important chemokine involved in neutrophil infiltration in humans. Chen et al. showed that IL-8 with Mp pneumonia was significantly increased compared to the controls and decreased in the convalescent phase (13). In addition, Mp induced the production of IL-8 in the human bronchial epithelial cells *in vitro* (13, 35). However, Chen et al. showed that there is no clear relationship between the number of Mp in the BALF and the level of IL-8 in the BALF in Mp pneumonia patients, although patients with a high number of Mp had a significantly higher number of neutrophils in the BALF than the patients with a low number of Mp (13). These results indicated the possibility that not only IL-8 but also other chemokines might be crucial for neutrophil infiltration into the BALF and the importance of elucidating the precise mechanism of neutrophil infiltration into the BALF in Mp pneumonia. We showed that the TLR2-dependent production of both IL-1α and IL-12 p40 contributes to the infiltration of neutrophils into the BALF, although each cytokine alone did not induce sufficient neutrophil infiltration (Fig. 4, Fig. S6). In addition, both IL-1α and IL-12 p40 were found to lead to the production of CXCL1, but not CXCL2 (Fig. 4). In previous reports, IL-1α drives the neutrophil infiltration in the lungs following Aspergillus fumigatus (36) and Legionella pneumophila infections (37). On the other hand, IL-12 p40 is a subunit of IL-12 and IL-23, and Salvatore et al. have reported the possibility that IL-12 partially contributes to the generation of inflammatory responses during Mp infection (38, 39). In addition, IL-23 induces IL-17 production by stimulating the γδT cells and monocytes, and IL-17 generally contributes to neutrophil infiltration via CXCL1 production (40, 41). Wu et al. have reported that the IL-23-mediated IL-17 production by the Mp challenge partially contributes to the neutrophil infiltration into the BALF in mice (42). Therefore, it is important to clarify the mechanism of concerted action of both IL-1α and IL-12 p40. Further studies are needed to investigate the role of CXCL1 induced by both IL-1α and IL-12 p40 in neutrophil infiltration into the lungs.

Alveolar macrophages constitutively express IL-1α inside the cells, and the extracellular release of IL-1α caused by the death of the alveolar macrophages contributes to inflammation, including that of neutrophil infiltration (43–46). We observed the death of the alveolar macrophages in the BALF 3 h after the Mp challenge in mice (Fig. 5). In addition, the alveolar macrophages produced IL-1α after Mp treatment *in vitro* upon the death of the alveolar macrophages rather than when these alveolar macrophages were alive (Fig. 6). Therefore, Mp may induce the death of the alveolar macrophages following the release of IL-1α *in vivo*. In contrast, the alveolar macrophages also produced IL-1α and IL-12 p40 after Mp treatment *in vitro* when the alveolar macrophages were alive (Fig. 6), indicating that Mp can induce the IL-1α and IL-12 p40 production by enhancing mRNA transcription after Mp challenge *in vivo*. In addition, ROS was found to be crucial for the alveolar macrophage deaths and IL-1α and IL-12 p40 production *in vitro* (Fig. 6). Since inflammatory cytokine induction from the alveolar macrophages by the TLR2 ligand was reduced in the presence of BHA (Fig. S8), the production of ROS was speculated to be caused by the TLR2 ligand of Mp. Taken together, these data suggest that the TLR2-mediated ROS contributes to the production of IL-1α and IL-12 p40 from the alveolar macrophages after Mp treatment.

CARDS toxin is believed to be essential in the process of inflammation caused by Mp (27–29). The CARDS toxin activates the NLR-family, leucine-rich repeat protein 3 (NLRP3) inflammasome by catalyzing the ADP-ribosylation of NLRP3, followed by the production of IL-1β (26, 47). The number of neutrophils in the lungs has been reported to be lower in the NLRP3 KO mice than in the wild-type mice after the Mp challenge (47). Therefore, it highlights that IL-1β is an important cytokine in CARDS toxin-induced inflammation. A previous report showed that respiratory exposure to the rCARDS toxin induces the production of the proinflammatory cytokines and the chemokines, including IL-1α and IL-1β in the BALF of mice and baboons (27). Here, we showed that rCARDS toxin induces the neutrophil infiltration into the BALF via the TLR2-independent IL-1α production, but not IL-12 p40 (Fig. 8). Currently, the amount of CARDS toxin produced in the lung during Mp infection remains unknown. Many reports have used around 50 μg/mouse rCARDS toxin to examine the activity of rCARDS toxin in mice (27, 28). As per these reports, we used 50 μg/mouse rCARDS toxin for the mouse experiments. However, there exists a possibility that 50 μg/mouse rCARDS toxin might lead to an overestimation of the function of CARDS toxin during the Mp challenge. Therefore, the amount of Mp in the lung during the Mp challenge remains to be clarified, in order to use the specific dose of rCARDS toxin to examine the contribution of the CARDS toxin *in vivo*. On the other hand, the CARDS toxin was observed to contribute to neutrophil infiltration during the Mp challenge (Fig. 7), although the contribution of TLR2 in neutrophil infiltration by CARDS toxin during the Mp challenge was not clear (Fig. S9, S11). These results suggested that not only TLR2-dependent production of both IL-1α and IL-12 p40, but also the CARDS toxin plays a crucial role in inflammation in Mp pneumonia. Furthermore, in the present study, we showed the possibility that CARDS toxin contributes to the persistence of Mp in the BALF (Fig. 7c). Up to 10% of total CARDS toxin is located on the surface of Mp (23). Therefore, we speculated the possibility that CARDS toxin located on the surface of Mp facilitates the attachment and the persistence of Mp on epithelial cells, promoting the TLR2-dependent neutrophil infiltration by Mp. Further investigation is required to understand the exact contribution of CARDS toxin located on Mp in the inflammation induced by Mp and the persistence of Mp in the lung. To elucidate this, only rCARDS toxin and anti-CARDS toxin Ab were used in the present study, but CARDS toxin-deficient Mp may also be useful.

Our findings have implications for developing therapeutics and vaccines against Mp. For example, therapeutics with the ability to inhibit IL-1α and IL-12 p40 signaling would be useful in suppressing the aggravation of Mp pneumonia. Hence, devising an Ab to the CARDS toxin and vaccine using the CARDS toxin as a vaccine antigen would be an appropriate approach. Therefore, further studies are needed to verify whether our concept has been established in humans.

## MATERIALS AND METHODS

### Mice.

Specific pathogen-free BALB/c mice were purchased from SLC (Shizuoka, Japan). The TLR2-deficient BALB/c mice were obtained from the Oriental Bio Service (Kyoto, Japan). The mice were used at 6–16 weeks of age. They were housed in a room with a 12:12-h light:dark cycle (lights on, 8:00 am; lights off, 8:00 pm) and had unrestricted access to food and water. All the animal experiments were performed in accordance with Osaka University’s Institutional Guidelines for the Ethical Treatment of Animals (protocol number H26-01-1).

### Mp challenge.

The FH strain of Mp was purchased from the American Type Culture Collection (Manassas, VA, USA). Mp was cultured as described previously (48, 49). The mice were challenged intranasally with 6 × 10^7^ CFU Mp in 40 μl of PBS under anesthesia. As a control, the mice were treated intranasally with 40 μl of PBS under anesthesia. As a positive control to induce lung injury, the mice were treated intranasally with 40 μl of 100 μg lipopolysaccharide from Escherichia coli 055:B5 (Sigma-Aldrich, St. Louis, MO, USA) under anesthesia. After Mp challenge, the DNA copies of Mp, immune cells, and the level of cytokines in BALF obtained by lavaging the lung with 1.2 ml PBS were analyzed. The DNA copies of Mp in BALF were determined by real-time PCR analysis as described previously (48, 49).

### LDH assay.

BALF was centrifuged at 600 × *g* for 5 min, and 100 μl of the supernatant was analyzed using the Cytotoxicity LDH assay kit-WST (Dojindo, Kumamoto, Japan) in accordance with the manufacturer’s instructions.

### The number of immune cells in BALF.

BALF was centrifuged at 600 × *g* for 5 min, and the resulting cell pellets were used to identify the various immune cell subsets in BALF using flow cytometry (NovoCyte Flow Cytometer, ACEA Bioscience, San Diego, CA, USA) as described previously (48, 49). We defined the neutrophils as CD45^+^ Ly6G^+^ CD11b^+^ Siglec-F^–^ and alveolar macrophages as CD45^+^ Ly6G^–^ CD11c^+^ Siglec-F^+^.

### The levels of cytokines and chemokines in BALF.

BALF was centrifuged at 6,000 × *g* for 5 min, and the concentrations of IL-1α, IL-6, IL-12 p40, CXCL1, CXCL2, and MMP-9 in the supernatants were analyzed using commercial ELISA kits ([BioLegend, San Diego, CA, USA] for IL-1α, IL-6, and IL-12 p40; [R&D Systems, Minneapolis, MN, USA] for CXCL1, CXCL2, and MMP-9, according to the manufacturer’s instructions). In some experiments, the lung was homogenated and centrifuged, and the concentrations of IL-1β in the supernatant was analyzed using the commercial ELISA kit (BioLegend).

### The levels of cytokine mRNA in BALF.

After centrifugation of the BALF at 600 × *g* for 5 min, the total RNA was extracted from the cell pellets using a Fast Gene RNA Basic-kit (NIPPON Genetics Co., Ltd., Tokyo, Japan). cDNA was generated by reverse transcription of the total mRNA using the ReverTra Ace qPCR RT Master Mix (TOYOBO, Osaka, Japan). The levels of the cytokine mRNA in the BALF were determined by real-time PCR analysis (Light Cycler 480 SYBR green |, Roche). We used primers specific for IL-1α (forward, 5′-TGA AGA GAC GGC TGA GT-3′; reverse, 5′-TGG TAG GTG TAA GGT GCT GAT-3′), IL-6 (forward, 5′-TGA ACA ACG ATG CAC TTG CAG A-3′; reverse, 5′-TCT GTA TCT CTC TGA AGG ACT CTG GCT-3′), and GAPDH (forward, 5′-TGA CGT GCC TGG AGA AA-3′; reverse, 5′-AGT GTA GCC CAA GAT GCC CTT CAG-3′).

### Neutrophil depletion.

The mice were injected intraperitoneally with an anti-Ly6G Ab (100 μg/mouse, clone 1A8, BioLegend) or isotype control Ab (100 μg/mouse, clone: RTK2758, BioLegend) on the day before the Mp challenge.

### Administration of anti-IL-1α Ab and/or anti-IL-12 p40 Ab.

The mice were injected intranasally with an anti-IL-1α Ab (20 μg/mouse, catalog numbers: AB-400-NA, R&D Systems) and/or anti-IL-12 p40 Ab (50 μg/mouse, clone: C17.8, Bio X Cell) 1 h before the Mp challenge. Goat IgG isotype Ab (20 μg/mouse, catalog numbers: AB-108-C, R&D Systems) was used as the control for anti-IL-1α Ab. Rat IgG2a isotype Ab (50 μg/mouse, clone: 2A3, Bio X Cell) was used as the control for anti-IL-12 p40 Ab.

### Death of the alveolar macrophages.

At 3 h after the Mp challenge, the viability of the alveolar macrophages in the BALF was analyzed using flow cytometry. In this study, we defined the death of the alveolar macrophages as 7AAD^+^ CD45^+^ Ly6G- CD11c^+^ Siglec-F^+^.

### Culture of the alveolar macrophages.

The cultured alveolar macrophages were prepared from the lung homogenates, as described previously (46). The alveolar macrophages (5 × 10^4^ cells/well) were cocultured with several doses of Mp (10^8^, 10^7^, or 10^6^ CFU/well) at 37°C for 24 h in the RPMI medium without antibiotics. The level of LDH in the culture supernatant was analyzed using the Cytotoxicity LDH assay kit-WST (Dojindo) in accordance with the manufacturer’s instructions. As a positive control for cell death, the alveolar macrophages were treated with lysis buffer included in the Cytotoxicity LDH assay kit (Dojindo). In some experiments, the alveolar macrophages were incubated with Mp and 150 μM BHA (Sigma-Aldrich) at 37°C for 24 h. In addition, the alveolar macrophages were incubated with 10 ng/ml Pam2CKS4 (InvivoGen, San Diego, CA, USA), a TLR2 ligand, with or without 150 μM BHA, at 37°C for 24 h.

### CARDS toxin.

The sequence of the CARDS toxin was derived from Mycoplasma pneumoniae M129 (GenBank accession number: NC_000912.1). The CARDS toxin plasmids (containing amino acids 1–591) with N-terminal hexahistidine tags were transformed into E. coli BL21(DE3). The expression of the rCARDS toxin protein was induced by adding 0.5 mM isopropyl-β-D-1-thiogalactopyranoside and then shaking for 16 h at 18°C. The rCARDS toxin was then purified by using an AKTA explorer chromatography system with a Ni-Sepharose HisTrap FF column (GE Healthcare, Diegem, Belgium). The rCARDS toxin was further purified using an EndoTrap HD column (LIONEX, Braunschweig, Germany) to remove the endotoxins. Additionally, to prevent aggregation, the rCARDS toxin was replaced with PBS containing 5% glycerol. The level of endotoxin in the rCARDS toxin (<0.05 EU/μg) was confirmed using the Limulus Color KY Test (Wako Pure Chemical Industries).

### CARDS toxin injection.

The mice were injected intranasally with the rCARDS toxin (50 μg/mouse) in 40 μl PBS under anesthesia. As a control, the mice were treated intranasally with 5% glycerol in 40 μl PBS under anesthesia. In some experiments, the mice were injected intranasally with an anti-IL-1α Ab and/or anti-IL-12 p40 Ab, or isotype control Ab 1 h before rCARDS toxin injection.

### Administration of the anti-CARDS toxin Ab.

Rabbit anti-CARDS toxin polyclonal Ab was obtained from Japan Bio Serum (Hiroshima, Japan). For the CARDS toxin depletion study, the mice were challenged intranasally with 6 × 10^7^ CFU Mp and anti-CARDS toxin polyclonal Ab (20 μg/mouse). At 24 h after the Mp challenge, the numbers of immune cells in BALF were analyzed. In another experiment, the mice were challenged intranasally with 6 × 10^7^ CFU Mp and anti-CARDS toxin Ab (20 μg/mouse) and then injected intranasally with the anti-CARDS toxin Ab again at 48 h after the Mp challenge. At 72 h after the Mp challenge, the numbers of immune cells in BALF were analyzed.

### Statistical analyses.

The statistical analyses were performed using the Prism software (GraphPad Software, San Diego, CA, USA). All data are presented as the mean ± standard deviation (SD). The significant differences were determined using Dunnett's test, Tukey’s test, or Student's *t* test. The statistical significance was set at *P* < 0.05.
