# Supplementary material for: Neutrophil-Mediated Lung Injury Both via TLR2-Dependent Production of IL-1α and IL-12 p40, and TLR2-Independent CARDS Toxin after Mycoplasma pneumoniae Infection in Mice
Source: Microbiol Spectr. 2021 Dec 22;9(3):e01588-21. doi: 10.1128/spectrum.01588-21 (PMC8694186; doi:10.1128/spectrum.01588-21)
Supplement: SUPPLEMENTAL FILE 1 — Supplemental material. Download SPECTRUM01588-21_Supp_1_seq12.pdf, PDF file, 0.4 MB [file spectrum01588-21_supp_1_seq12.pdf]

Materials and Methods

The vacuolating activity of the recombinant CARDS toxin

The A549 (human lung adenocarcinoma cell line) cells were obtained from the American Type Culture Collection (Manassas, VA) and A549 cells (1×10^4 cells/well) were added to a 96-well plate and grown for 24 h at 37°C. After removing the culture medium and washing the cells with PBS, the A549 cells were treated with the rCARDS toxin (0, 50, or 100 μg/mL) in the absence of the FCS, and the FCS (final 10%) was added 2 h later. After 48 h of incubation with the rCARDS toxin, the number of vacuolations in the A549 cells was observed using microscopy.

Induction of IL-1β by the recombinant CARDS toxin

The THP-1 (human acute monocytic leukemia cell line) cells were obtained from the American Type Culture Collection and cultured at 37°C in RPMI supplemented with 10% FCS, 2 mM l-glutamine, and antibiotics. The THP-1 cells (3×10^4 cells/well) were seeded in the 96-well plates and differentiated into macrophages by incubation with 0.5 μM phorbol 12-myristate 13-acetate (PMA, Sigma-Aldrich) at 37°C for 24 h. After PMA priming, the cells were treated with 100 μg/mL of rCARDS toxin with or without 30 μM Ac-YVAD-CHO (Cayman Chemical, Ann Arbor, MI) at 37°C in RPMI without FCS. After 2 h, FCS (final 10%) was added and the cells were incubated for 24 h. The IL-1β in the supernatant was assessed using a human IL-1β ELISA kit (catalog number: DY201-05, R&D Systems).

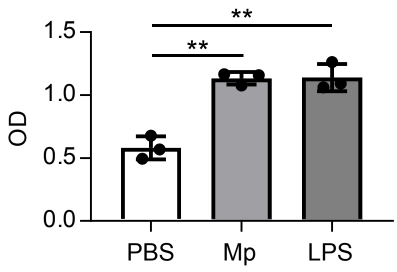

Figure S1. Lung injury after the Mp challenge and LPS treatment. Related to Figure 1. The mice were challenged intranasally with Mp (6 × 10^7 CFU). As a control, the mice were treated with 100 μg lipopolysaccharide (LPS) intranasally. At 24 h after the Mp challenge or LPS treatment, the level of LDH in the BALF was measured. Each experiment was performed more than twice. Data are shown as means ± SD. n = 3; $^{**}P < 0.01$ as indicated by Tukey's test.

Supplementary Figure S1

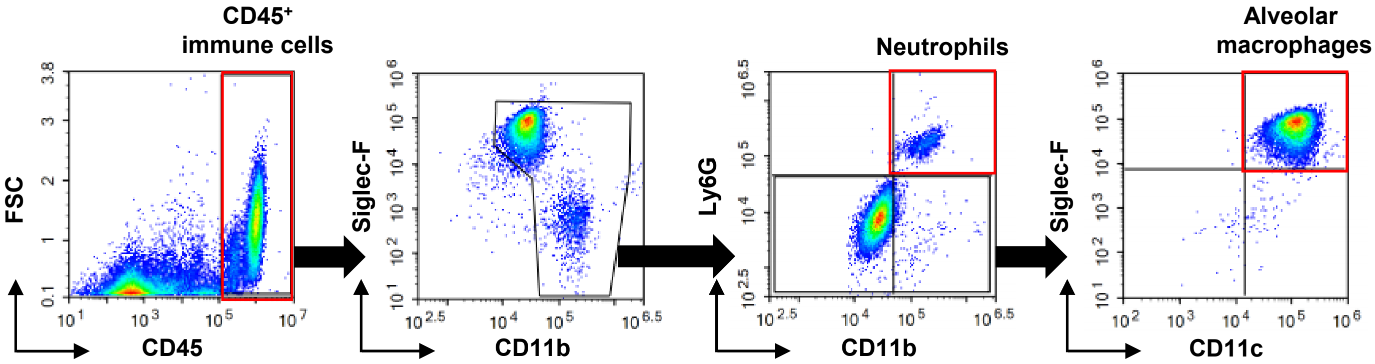

Figure S2. Flow cytometry. Related to Figure 1. A gating strategy for analyzing the BALF by flow cytometry has been shown. The numbers of CD45$^+$ immune cells, neutrophils (CD45$^+$ Ly6G$^+$ CD11b$^+$ Siglec-F$^-$) and alveolar macrophages (CD45$^+$ Ly6G$^-$ CD11c$^+$ Siglec-F$^+$) in the BALF were measured.

Supplementary Figure S2

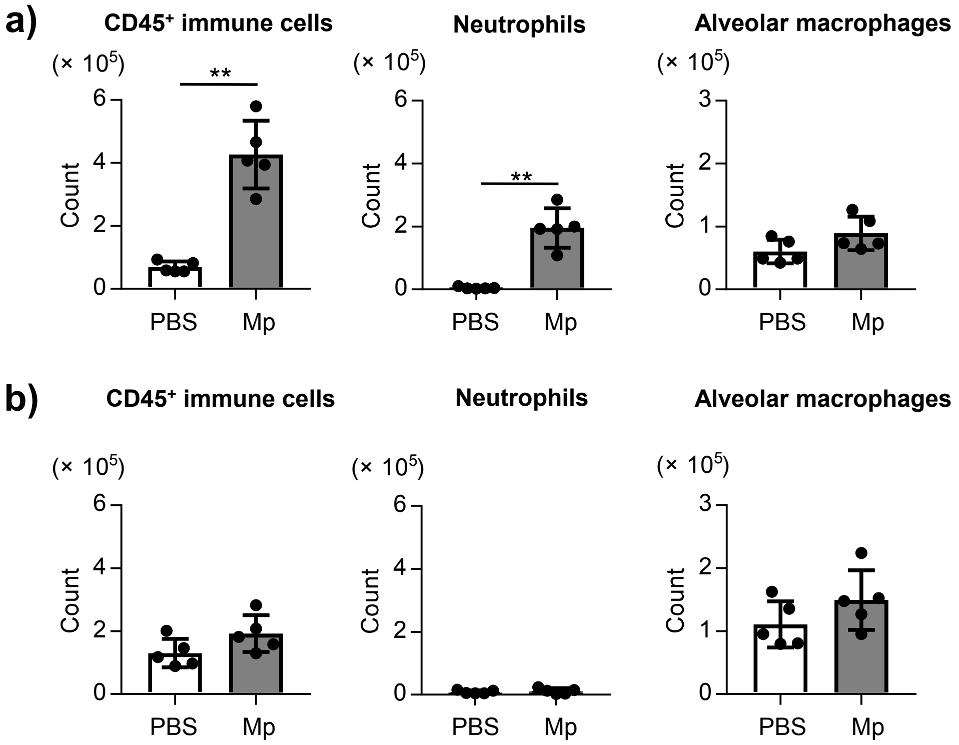

Figure S3. The inflammation in the lungs after the Mp challenge. Related to Figure 1. The mice were challenged intranasally with Mp (6×10^7 CFU). As a control, the mice were treated with PBS intranasally. At (a) 72 h and (b) 120 h after Mp challenge, the numbers of $CD45^+$ immune cells, neutrophils ($CD45^+$ $Ly6G^+$ $CD11b^+$ Siglec-F^-), and alveolar macrophages ($CD45^+$ $Ly6G^-$ $CD11c^+$ Siglec-F^+) in the BALF were measured by flow cytometry. (a, b) Each experiment was performed more than twice. Data are shown as means ± SD. $n = 5$. (a) $^{**}P < 0.01$ as indicated by Student's *t*-test.

Supplementary Figure S3

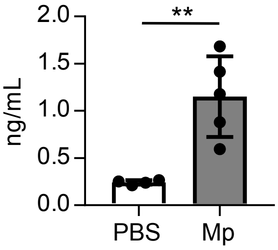

Figure S4. Cytokine production in lung after the Mp challenge. Related to Figure 1. The mice were challenged intranasally with Mp (6×10^7 CFU). As a control, the mice were treated with PBS intranasally. At 24 h after the Mp challenge, the level of IL-1β in lung homogenate was measured by ELISA. Each experiment was performed more than twice. Data are shown as means ± SD. *n* = 4–5. $^{**}P < 0.01$ as indicated by Student's *t*-test.

Supplementary Figure S4

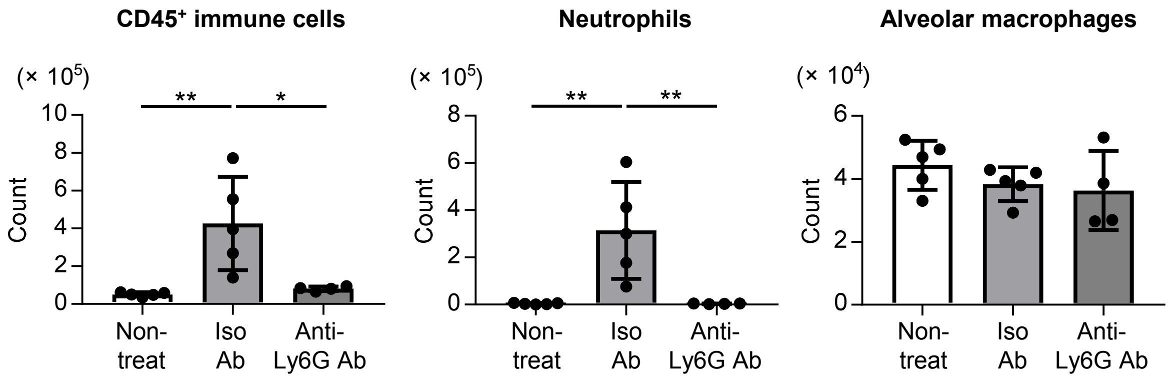

Figure S5. Neutrophil depletion by using anti-Ly6G antibody. Related to Figure 2. The mice were treated intraperitoneally with an anti-Ly6G antibody (Ab) (100 μg/mouse) or isotype control Ab, and challenged with Mp (6×10^7 CFU) on 24 h after Ab treatment. Non-treat group indicated mice treated with PBS intranasally and without Ab treatment. On 24 h after the Mp challenge, the numbers of $CD45^+$ immune cells, the neutrophils ($CD45^+$ $Ly6G^+$ $CD11b^+$ Siglec-F^-), and alveolar macrophages ($CD45^+$ $Ly6G^-$ $CD11c^+$ Siglec-F^+) in the BALF were measured by flow cytometry. Each experiment was performed more than twice. Data are shown as means ± SD. n = 4–5. *P < 0.05; **P < 0.01 as indicated by Tukey's test.

Supplementary Figure S5

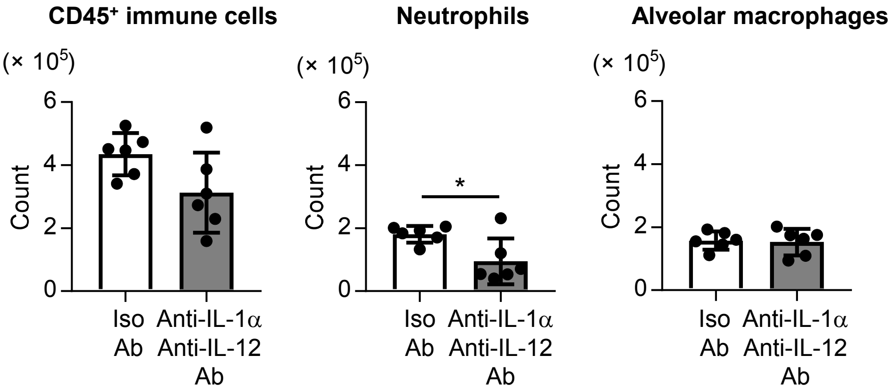

Figure S6. The contribution of both IL-1α and IL-12 p40 for the Mp-mediated lung injury and inflammation. Related to Figure 4. The mice were treated intranasally with an anti-IL-1α antibody (Ab) (20 μg/mouse) and/or an anti-IL-12 p40 Ab (50 μg/mouse), or isotype control Ab, and challenged with Mp (6×10^7 CFU) on 1 h after Ab treatment. At 72 h after the Mp challenge, the numbers of $CD45^+$ immune cells, neutrophils ($CD45^+$ $Ly6G^+$ $CD11b^+$ Siglec-F^-), and alveolar macrophages ($CD45^+$ $Ly6G^-$ $CD11c^+$ Siglec-F^+) in the BALF were measured by flow cytometry. Each experiment was performed more than twice. Data are shown as means ± SD. $n = 6$. $^*P < 0.05$ as indicated by Student's *t*-test.

Supplementary Figure S6

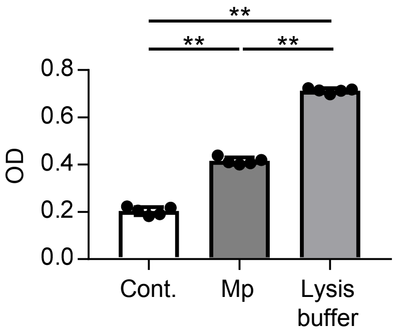

Figure S7. Death of alveolar macrophages by Mp in vitro. Related to Figure 6. The alveolar macrophages were treated with Mp (1×10^8 CFU/well) for 24 h. As a positive control for cell death, the alveolar macrophages were treated with lysis buffer. The LDH level in the culture supernatant was measured to evaluate the cytotoxic effect of Mp. Each experiment was performed more than twice. Data are shown as means ± SD. $n = 5$; $^{**}P < 0.01$ as indicated by Tukey's test.

Supplementary Figure S7

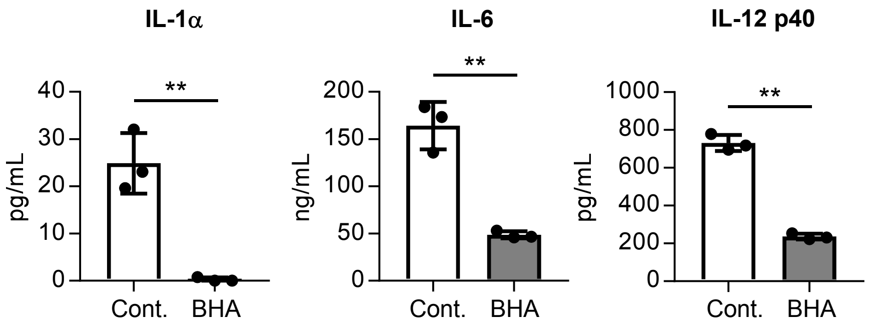

Figure S8. Role of ROS in the TLR2-mediated production of the inflammatory cytokines. Related to Figure 6. The alveolar macrophages were treated with Pam2CKS4, a TLR2 ligand, for 24 h in the absence or presence of BHA, ROS inhibitor. The levels of IL-1α, IL-6, and IL-12 p40 in the culture supernatant were measured by ELISA. Each experiment was performed more than twice. Data are shown as means ± SD. n = 3. **P < 0.01 as indicated by Student's t-test.

Supplementary Figure S8

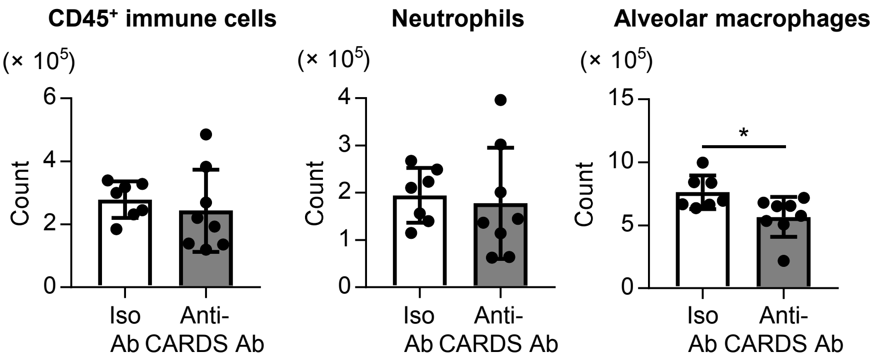

Figure S9. The effect of anti-CARDS toxin Ab in TLR2-deficient mice. **Related to Figure 7.** The *TLR2*$^{-/-}$ mice were challenged intranasally with Mp (6×10^7 CFU) plus anti-CARDS toxin polyclonal antibody (Ab) or isotype control Ab. After 24 h, the numbers of CD45$^+$ immune cells, neutrophils (CD45$^+$ Ly6G$^+$ CD11b$^+$ Siglec-F$^-$), and alveolar macrophages (CD45$^+$ Ly6G$^-$ CD11c$^+$ Siglec-F$^+$) in the BALF were measured by flow cytometry. Each experiment was performed more than twice. Data are shown as means ± SD. n = 7–8. *P < 0.05 as indicated by the Student's t-test.

Supplementary Figure S9

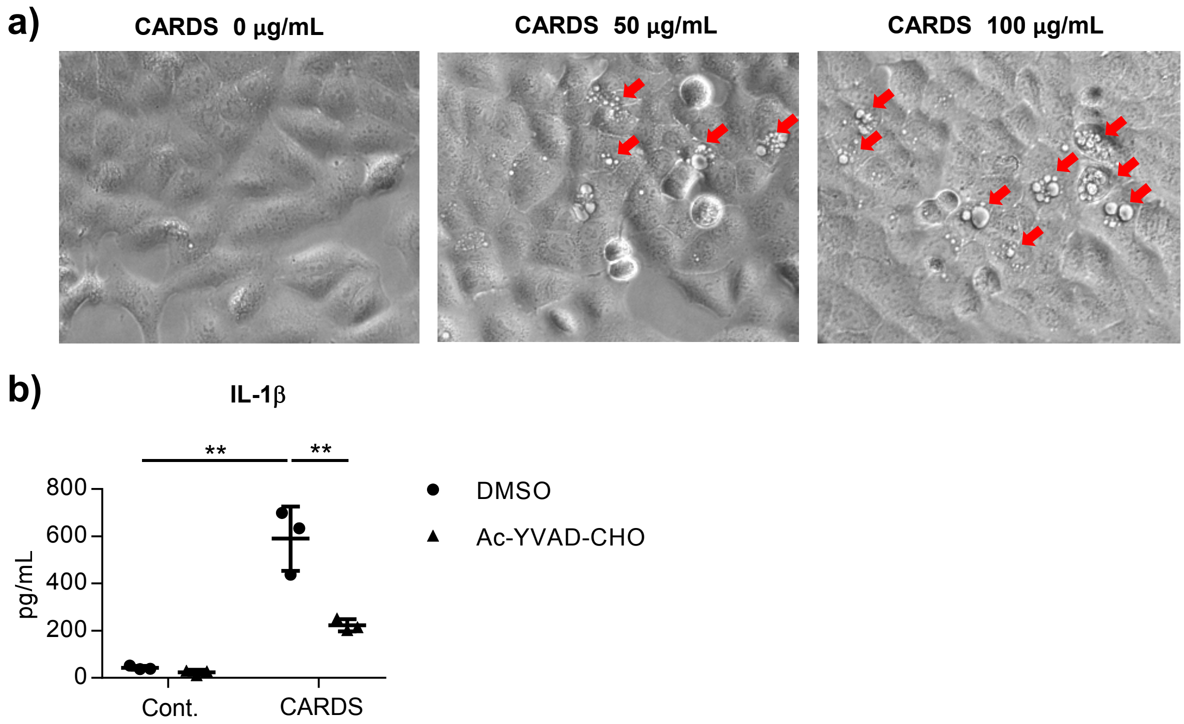

Figure S10. Validation of the recombinant CARDS toxin. Related to Figure 8. (a) A549 cells (1 × 10^4 cells/well) were treated with rCARDS toxin (0, 50, 100 μg/mL) at 37°C. At 48 h after rCARDS toxin treatment, the vacuolization of the A549 cells was evaluated by microscopy. (b) PMA-differentiated THP-1 cells (3 × 10^4 cells/well) were treated with a caspase-1 inhibitor, Ac-YVAD-CHO, and rCARDS toxin (100 μg/mL) at 37°C. At 24 h after treatment, the level of IL-1β in the supernatant was measured by ELISA. (a, b) Each experiment was performed more than twice. (b) Data are shown as means ± SD. $n = 3$. $^{**}P < 0.01$ as indicated by Tukey's test.

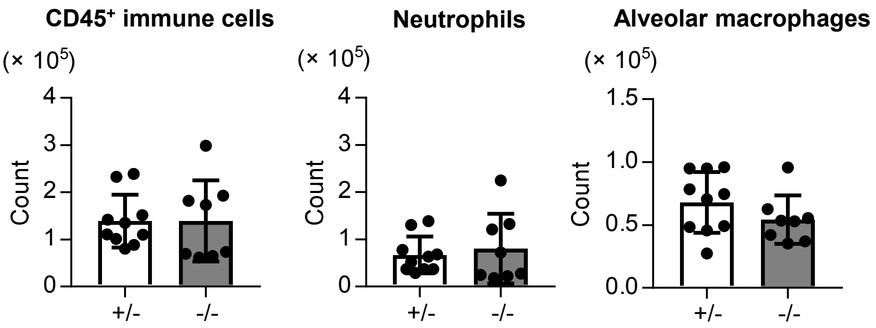

Figure S11. The dispensable role of TLR2 in neutrophil recruitment by CARDS toxin. Related to Figure 8. *TLR2*$^{+/-}$ and *TLR2*$^{-/-}$ mice were challenged intranasally with rCARDS toxin (50 μg/mouse). At 24 h after rCARDS toxin treatment, the numbers of CD45^{+} immune cells, neutrophils (CD45^{+} Ly6G^{+} CD11b^{+} Siglec-F^{-}), and alveolar macrophages (CD45^{+} Ly6G^{-} CD11c^{+} Siglec-F^{+}) in the BALF were measured by flow cytometry. Each experiment was performed more than twice. *n* = 8–10.

Supplementary Figure S11
